# 2-[2-Hydr­oxy-4-(pyrrolidin-1-yl)benzo­yl]benzoic acid

**DOI:** 10.1107/S1600536809011349

**Published:** 2009-03-31

**Authors:** Yun-Long Gao, Jian-Wu Wang

**Affiliations:** a School of Chemistry and Chemical Engineering, Shandong University, Jinan 250100, People’s Republic of China

## Abstract

The title compound, C_18_H_17_NO_4_, crystallizes with two indepen­dent mol­ecules in the asymmetric unit. The pyrrolidine ring in one mol­ecule is disordered over two positions, with refined site-occupancy factors of 0.853 (5) and 0.147 (5). The dihedral angles between the planes of the benzene rings in the two independent mol­ecules are 56.8 (2) and 68.2 (5)°. The mol­ecular conformations are stabilized by intra­molecular O—H⋯O hydrogen bonds. In the crystal structure, mol­ecules are linked by inter­molecular O—H⋯O hydrogen bonds, forming dimers and generating rings of graph-set motif *R*_2_^2^(8).

## Related literature

For the synthesis and applications of the title compound, see: Lee *et al.* (2005 ▶); Masakichi *et al.* (1974 ▶); Luo *et al.* (1994 ▶). For bond-length and angle data for pyrrolidines, see: Effenberger *et al.* (1983 ▶). For hydrogen-bond motifs, see: Bernstein *et al.* (1995 ▶).
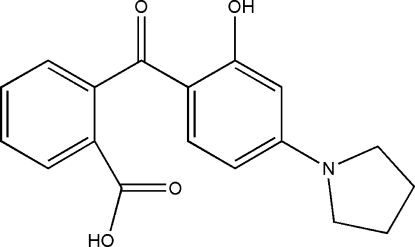


## Experimental

### 

#### Crystal data

C_18_H_17_NO_4_
*M* *_r_* = 311.33
Triclinic, 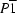

*a* = 10.841 (2) Å
*b* = 11.878 (2) Å
*c* = 13.781 (3) Å
α = 71.70 (3)°
β = 82.05 (3)°
γ = 65.17 (3)°
*V* = 1529.0 (7) Å^3^
*Z* = 4
Mo *K*α radiation
μ = 0.10 mm^−1^
*T* = 113 K
0.18 × 0.16 × 0.12 mm

#### Data collection

Rigaku SATURN CCD area-detector diffractometer
Absorption correction: multi-scan (*CrystalClear*; Rigaku, 2005 ▶) *T*_min_ = 0.983, *T*_max_ = 0.989
13535 measured reflections
6888 independent reflections
4673 reflections with *I* > 2σ(*I*)
*R*_int_ = 0.036

#### Refinement

*R*[*F* ^2^ > 2σ(*F* ^2^)] = 0.048
*wR*(*F* ^2^) = 0.136
*S* = 1.04
6888 reflections
437 parameters
10 restraints
H atoms treated by a mixture of independent and constrained refinement
Δρ_max_ = 0.33 e Å^−3^
Δρ_min_ = −0.25 e Å^−3^

### 

Data collection: *CrystalClear* (Rigaku, 2005 ▶); cell refinement: *CrystalClear*; data reduction: *CrystalClear*; program(s) used to solve structure: *SHELXTL* (Sheldrick, 2008 ▶); program(s) used to refine structure: *SHELXTL*; molecular graphics: *SHELXTL*; software used to prepare material for publication: *SHELXTL*.

## Supplementary Material

Crystal structure: contains datablocks I, global. DOI: 10.1107/S1600536809011349/rz2305sup1.cif

Structure factors: contains datablocks I. DOI: 10.1107/S1600536809011349/rz2305Isup2.hkl

Additional supplementary materials:  crystallographic information; 3D view; checkCIF report

## Figures and Tables

**Table 1:** Hydrogen-bond geometry (Å, °)

*D*—H⋯*A*	*D*—H	H⋯*A*	*D*⋯*A*	*D*—H⋯*A*
O1—H1⋯O2	0.956 (19)	1.66 (2)	2.547 (2)	151.8 (18)
O5—H5⋯O6	0.943 (19)	1.68 (2)	2.565 (2)	154.9 (19)
O7—H7*A*⋯O3^i^	0.86 (2)	1.784 (10)	2.6387 (17)	169.3 (19)
O4—H4⋯O8^ii^	0.879 (10)	1.785 (11)	2.6451 (19)	166 (2)

Symmetry codes: (i) 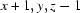; (ii) 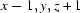.

**Fig. 1.**
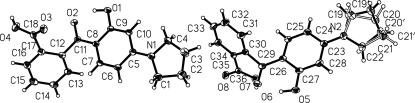
View of the title compound, with displacement ellipsoids drawn at the 40% probability level.

## supplementary crystallographic information

### Comment

2-[2-Hydroxy-4-(1-pyrrolidinyl)benzoyl)]benzoic acid is an intermediate in the synthesis of pyrrolidinylrhodamine (Lee *et al.*, 2005) and its derivatives (Masakichi *et al.*, 1974). It has been synthesized from 3-pyrrolidinylphenol and phthalic anhydride in toluene (Luo *et al.*,1994). Although its synthesis has been studied, the crystal structure of title compound has not been investigated. In this paper we reported its crystal structure.

The title compound crystallizes with two independent molecules in the asymmetric unit (Fig. 1). Bond lengths and angles within the pyrrolidine rings are normal and in good agreement with those reported previously for 2,4,6-tripyrrolidino-2',4',6'-trinitrobiphenyl (Effenberger *et al.*, 1983). The dihedral angles between the planes of the benzene rings in the two independent molecules are 56.8 (2) and 68.2 (5)°. The molecular conformations are stabilized by intramolecular O—H···O hydrogen bonds (Table 1). In the crystal packing, the molecules are linked by intermolecular O—H···O hydrogen bonds to form dimers generating rings of graph-set motif *R*_2_^2^(8) (Bernstein *et al.*, 1995).

### Experimental

A solution of 3-pyrrolidinylphenol (1.20 g, 7.36 mmol) and phthalic anhydride (1.31 g, 8.83 mmol) in toluene was refluxed under N_2_ for 3 h. The mixture was cooled to 50–60°C. Then 7 ml of 35.0% aqueous NaOH (*w*/*w*) was added and heated at 90° C for 6 h. The resulting mixture was poured into 70 ml of H2O, acidified with hydrochloric acid, and allowed to stand at room temperature for 2 h. The suspension was then filtered. The solid was recrystallized from a mixture of water and methanol, and then dried to afford the desired product (1.63 g, 70.7%). Crystals suitable for X-ray diffraction were obtained by slow evaporation of a CD_3_OD/CDCl_3_ (5:1 *v*/*v*) solution.

### Refinement

Hydroxy H atoms were found on a difference Fourier map and isotropically refined with *U*_iso_(H) = 1.5 *U*_eq_(O). All other H atoms were placed at calculated positions and refined using a riding model, with C—H = 0.95–0.99 Å and with *U*_iso_(H) = 1.2*U*_eq_(C). The pyrrolidine group in one molecular was found to be disordered. Atoms C19, C20 and C21 were therefore refined over two positions with refined occupancies of 0.853 (5) and 0.147 (5) for primed and unprimed atoms, respectively.

### Crystal data

**Table:**

C_18_H_17_NO_4_	*Z* = 4
*M**_r_* = 311.33	*F*(000) = 656
Triclinic, *P*1	*D*_x_ = 1.352 Mg m^−^^3^
Hall symbol: -P 1	Mo *K*α radiation, λ = 0.71073 Å
*a* = 10.841 (2) Å	Cell parameters from 4028 reflections
*b* = 11.878 (2) Å	θ = 1.6–27.5°
*c* = 13.781 (3) Å	µ = 0.10 mm^−^^1^
α = 71.70 (3)°	*T* = 113 K
β = 82.05 (3)°	Block, colourless
γ = 65.17 (3)°	0.18 × 0.16 × 0.12 mm
*V* = 1529.0 (7) Å^3^	

### Data collection

**Table:**

Rigaku SATURN CCD area-detector diffractometer	6888 independent reflections
Radiation source: rotating anode	4673 reflections with *I* > 2σ(*I*)
confocal	*R*_int_ = 0.036
Detector resolution: 7.31 pixels mm^-1^	θ_max_ = 27.5°, θ_min_ = 1.6°
ω and φ scans	*h* = −14→10
Absorption correction: multi-scan (*CrystalClear*; Rigaku, 2005)	*k* = −15→13
*T*_min_ = 0.983, *T*_max_ = 0.989	*l* = −17→16
13535 measured reflections	

### Refinement

**Table:**

Refinement on *F*^2^	Primary atom site location: structure-invariant direct methods
Least-squares matrix: full	Secondary atom site location: difference Fourier map
*R*[*F*^2^ > 2σ(*F*^2^)] = 0.048	Hydrogen site location: inferred from neighbouring sites
*wR*(*F*^2^) = 0.136	H atoms treated by a mixture of independent and constrained refinement
*S* = 1.04	*w* = 1/[σ^2^(*F*_o_^2^) + (0.0749*P*)^2^] where *P* = (*F*_o_^2^ + 2*F*_c_^2^)/3
6888 reflections	(Δ/σ)_max_ < 0.001
437 parameters	Δρ_max_ = 0.33 e Å^−^^3^
10 restraints	Δρ_min_ = −0.25 e Å^−^^3^

### Special details

**Table:**

Geometry. All e.s.d.'s (except the e.s.d. in the dihedral angle between two l.s. planes) are estimated using the full covariance matrix. The cell e.s.d.'s are taken into account individually in the estimation of e.s.d.'s in distances, angles and torsion angles; correlations between e.s.d.'s in cell parameters are only used when they are defined by crystal symmetry. An approximate (isotropic) treatment of cell e.s.d.'s is used for estimating e.s.d.'s involving l.s. planes.
Refinement. Refinement of *F*^2^ against ALL reflections. The weighted *R*-factor *wR* and goodness of fit *S* are based on *F*^2^, conventional *R*-factors *R* are based on *F*, with *F* set to zero for negative *F*^2^. The threshold expression of *F*^2^ > σ(*F*^2^) is used only for calculating *R*-factors(gt) *etc*. and is not relevant to the choice of reflections for refinement. *R*-factors based on *F*^2^ are statistically about twice as large as those based on *F*, and *R*- factors based on ALL data will be even larger.

### Fractional atomic coordinates and isotropic or equivalent isotropic displacement parameters (Å^2^)

**Table:**

	*x*	*y*	*z*	*U*_iso_*/*U*_eq_	Occ. (<1)
O1	0.45366 (12)	0.59128 (10)	1.11419 (8)	0.0288 (3)	
H1	0.443 (2)	0.6119 (18)	1.1774 (14)	0.043*	
O2	0.40164 (12)	0.71748 (10)	1.24448 (8)	0.0294 (3)	
O3	0.09923 (13)	0.84631 (12)	1.27107 (8)	0.0348 (3)	
O4	0.10829 (15)	0.86955 (13)	1.42445 (9)	0.0429 (3)	
H4	0.053 (2)	0.829 (2)	1.4387 (16)	0.064*	
O5	0.67090 (12)	0.78113 (10)	0.09493 (8)	0.0318 (3)	
H5	0.653 (2)	0.8114 (19)	0.1531 (15)	0.048*	
O6	0.66160 (12)	0.79633 (10)	0.27761 (8)	0.0308 (3)	
O7	0.93501 (12)	0.72631 (11)	0.34563 (8)	0.0293 (3)	
H7A	0.9825 (18)	0.7726 (17)	0.3252 (14)	0.044*	
O8	0.94767 (13)	0.74504 (12)	0.49982 (8)	0.0371 (3)	
N1	0.34912 (15)	0.82293 (12)	0.76564 (9)	0.0292 (3)	
N2	0.90372 (15)	0.36958 (13)	0.03616 (10)	0.0304 (3)	
C1	0.3095 (2)	0.94066 (17)	0.67866 (12)	0.0426 (5)	
H1A	0.3482	1.0003	0.6846	0.051*	
H1B	0.2094	0.9865	0.6733	0.051*	
C2	0.3704 (3)	0.88786 (19)	0.58723 (14)	0.0582 (6)	
H2A	0.3169	0.9446	0.5246	0.070*	
H2B	0.4658	0.8789	0.5746	0.070*	
C3	0.3620 (2)	0.75708 (18)	0.62026 (13)	0.0483 (5)	
H3A	0.4273	0.6993	0.5812	0.058*	
H3B	0.2692	0.7660	0.6107	0.058*	
C4	0.3988 (2)	0.70541 (15)	0.73310 (12)	0.0330 (4)	
H4A	0.3530	0.6478	0.7715	0.040*	
H4B	0.4982	0.6575	0.7425	0.040*	
C5	0.34975 (16)	0.82278 (15)	0.86414 (11)	0.0245 (3)	
C6	0.29963 (17)	0.94070 (14)	0.89045 (11)	0.0265 (3)	
H6	0.2632	1.0209	0.8390	0.032*	
C7	0.30356 (16)	0.93921 (14)	0.98950 (11)	0.0243 (3)	
H7	0.2693	1.0193	1.0053	0.029*	
C8	0.35634 (16)	0.82382 (14)	1.06903 (11)	0.0221 (3)	
C9	0.40463 (16)	0.70647 (14)	1.04206 (11)	0.0228 (3)	
C10	0.40242 (16)	0.70541 (14)	0.94228 (11)	0.0240 (3)	
H10	0.4365	0.6254	0.9263	0.029*	
C11	0.35830 (16)	0.82146 (14)	1.17426 (11)	0.0236 (3)	
C12	0.31291 (16)	0.94406 (14)	1.20471 (11)	0.0240 (3)	
C13	0.37622 (17)	1.02936 (15)	1.16136 (12)	0.0275 (4)	
H13	0.4382	1.0162	1.1060	0.033*	
C14	0.34921 (18)	1.13357 (15)	1.19853 (13)	0.0325 (4)	
H14	0.3918	1.1919	1.1679	0.039*	
C15	0.26080 (19)	1.15270 (16)	1.27972 (13)	0.0349 (4)	
H15	0.2442	1.2230	1.3058	0.042*	
C16	0.19610 (18)	1.06921 (15)	1.32333 (12)	0.0307 (4)	
H16	0.1347	1.0829	1.3789	0.037*	
C17	0.22088 (17)	0.96580 (15)	1.28596 (11)	0.0254 (3)	
C18	0.13923 (17)	0.88714 (15)	1.32972 (11)	0.0264 (3)	
C19	0.9822 (5)	0.2294 (2)	0.0686 (2)	0.0342 (8)	0.853 (5)
H19A	0.9480	0.1890	0.1347	0.041*	0.853 (5)
H19B	1.0797	0.2085	0.0755	0.041*	0.853 (5)
C20	0.9604 (2)	0.1837 (2)	−0.01711 (17)	0.0335 (6)	0.853 (5)
H20A	0.8789	0.1633	−0.0039	0.040*	0.853 (5)
H20B	1.0406	0.1065	−0.0258	0.040*	0.853 (5)
C21	0.9410 (3)	0.3008 (3)	−0.1104 (2)	0.0376 (7)	0.853 (5)
H21A	1.0300	0.2986	−0.1406	0.045*	0.853 (5)
H21B	0.8867	0.3016	−0.1630	0.045*	0.853 (5)
C19'	0.967 (4)	0.2271 (10)	0.0567 (12)	0.0342 (8)	0.147 (5)
H19C	0.9022	0.1871	0.0880	0.041*	0.147 (5)
H19D	1.0482	0.1871	0.1009	0.041*	0.147 (5)
C20'	1.0060 (14)	0.2181 (13)	−0.0526 (10)	0.0335 (6)	0.147 (5)
H20C	1.0950	0.2245	−0.0703	0.040*	0.147 (5)
H20D	1.0166	0.1323	−0.0556	0.040*	0.147 (5)
C21'	0.903 (2)	0.3222 (14)	−0.1313 (13)	0.0376 (7)	0.147 (5)
H21C	0.8261	0.3005	−0.1377	0.045*	0.147 (5)
H21D	0.9437	0.3472	−0.1991	0.045*	0.147 (5)
C22	0.8662 (2)	0.42004 (18)	−0.07185 (12)	0.0351 (4)	
H22A	0.8965	0.4877	−0.1071	0.042*	0.853 (5)
H22B	0.7697	0.4524	−0.0797	0.042*	0.853 (5)
H22C	0.9083	0.4791	−0.1054	0.042*	0.147 (5)
H22D	0.7696	0.4691	−0.0761	0.042*	0.147 (5)
C23	0.86444 (17)	0.44414 (15)	0.10045 (11)	0.0257 (3)	
C24	0.90176 (18)	0.38879 (15)	0.20557 (11)	0.0286 (4)	
H24	0.9581	0.2993	0.2301	0.034*	
C25	0.85731 (17)	0.46321 (14)	0.27076 (11)	0.0258 (3)	
H25	0.8821	0.4238	0.3407	0.031*	
C26	0.77521 (16)	0.59747 (14)	0.23827 (11)	0.0235 (3)	
C27	0.74369 (16)	0.65237 (14)	0.13248 (11)	0.0244 (3)	
C28	0.78626 (17)	0.57779 (15)	0.06598 (11)	0.0269 (4)	
H28	0.7625	0.6171	−0.0041	0.032*	
C29	0.72606 (16)	0.67720 (14)	0.30674 (11)	0.0241 (3)	
C30	0.74358 (16)	0.61217 (14)	0.42053 (11)	0.0246 (3)	
C31	0.67243 (18)	0.53419 (16)	0.46469 (12)	0.0323 (4)	
H31	0.6218	0.5196	0.4225	0.039*	
C32	0.67458 (18)	0.47761 (17)	0.56943 (13)	0.0354 (4)	
H32	0.6259	0.4243	0.5985	0.043*	
C33	0.74738 (18)	0.49875 (16)	0.63124 (12)	0.0346 (4)	
H33	0.7481	0.4608	0.7031	0.042*	
C34	0.81990 (17)	0.57562 (15)	0.58857 (11)	0.0296 (4)	
H34	0.8701	0.5898	0.6315	0.036*	
C35	0.81935 (17)	0.63226 (14)	0.48276 (11)	0.0244 (3)	
C36	0.90522 (16)	0.70674 (14)	0.43999 (11)	0.0243 (3)	

### Atomic displacement parameters (Å^2^)

**Table:**

	*U*^11^	*U*^22^	*U*^33^	*U*^12^	*U*^13^	*U*^23^
O1	0.0362 (7)	0.0172 (5)	0.0278 (6)	−0.0074 (5)	−0.0014 (5)	−0.0039 (4)
O2	0.0368 (7)	0.0223 (6)	0.0260 (5)	−0.0121 (5)	0.0023 (5)	−0.0034 (4)
O3	0.0379 (8)	0.0426 (7)	0.0374 (6)	−0.0250 (6)	0.0079 (5)	−0.0202 (5)
O4	0.0558 (10)	0.0545 (9)	0.0291 (6)	−0.0343 (7)	0.0099 (6)	−0.0132 (6)
O5	0.0383 (8)	0.0202 (6)	0.0303 (6)	−0.0079 (5)	−0.0105 (5)	0.0007 (4)
O6	0.0342 (7)	0.0200 (6)	0.0348 (6)	−0.0087 (5)	−0.0039 (5)	−0.0048 (5)
O7	0.0327 (7)	0.0353 (7)	0.0248 (5)	−0.0196 (6)	0.0058 (5)	−0.0093 (5)
O8	0.0495 (9)	0.0444 (7)	0.0277 (6)	−0.0267 (7)	0.0010 (5)	−0.0132 (5)
N1	0.0357 (9)	0.0216 (7)	0.0271 (7)	−0.0082 (6)	−0.0030 (6)	−0.0063 (5)
N2	0.0358 (9)	0.0307 (7)	0.0286 (7)	−0.0152 (7)	−0.0003 (6)	−0.0110 (6)
C1	0.0649 (15)	0.0269 (9)	0.0294 (8)	−0.0128 (9)	−0.0119 (8)	−0.0022 (7)
C2	0.095 (2)	0.0420 (11)	0.0305 (9)	−0.0216 (12)	−0.0078 (10)	−0.0060 (8)
C3	0.0655 (16)	0.0352 (10)	0.0351 (9)	−0.0070 (10)	−0.0115 (9)	−0.0118 (8)
C4	0.0399 (11)	0.0242 (8)	0.0322 (8)	−0.0077 (8)	−0.0037 (7)	−0.0101 (7)
C5	0.0217 (9)	0.0231 (8)	0.0283 (7)	−0.0090 (7)	0.0019 (6)	−0.0075 (6)
C6	0.0286 (10)	0.0176 (7)	0.0288 (7)	−0.0073 (7)	−0.0022 (6)	−0.0026 (6)
C7	0.0235 (9)	0.0189 (7)	0.0296 (7)	−0.0082 (7)	0.0026 (6)	−0.0074 (6)
C8	0.0196 (8)	0.0195 (7)	0.0272 (7)	−0.0095 (7)	0.0022 (6)	−0.0052 (6)
C9	0.0202 (8)	0.0166 (7)	0.0283 (7)	−0.0070 (7)	0.0021 (6)	−0.0035 (6)
C10	0.0247 (9)	0.0179 (7)	0.0287 (7)	−0.0086 (7)	0.0023 (6)	−0.0066 (6)
C11	0.0206 (9)	0.0200 (7)	0.0287 (7)	−0.0094 (7)	0.0027 (6)	−0.0044 (6)
C12	0.0248 (9)	0.0215 (7)	0.0260 (7)	−0.0092 (7)	−0.0048 (6)	−0.0054 (6)
C13	0.0243 (9)	0.0237 (8)	0.0329 (8)	−0.0095 (7)	−0.0033 (6)	−0.0051 (6)
C14	0.0311 (10)	0.0237 (8)	0.0435 (9)	−0.0127 (8)	−0.0080 (8)	−0.0049 (7)
C15	0.0391 (11)	0.0268 (9)	0.0429 (9)	−0.0118 (8)	−0.0093 (8)	−0.0135 (7)
C16	0.0323 (10)	0.0287 (9)	0.0306 (8)	−0.0085 (8)	−0.0029 (7)	−0.0117 (7)
C17	0.0271 (9)	0.0241 (8)	0.0248 (7)	−0.0095 (7)	−0.0043 (6)	−0.0058 (6)
C18	0.0279 (10)	0.0258 (8)	0.0241 (7)	−0.0083 (7)	0.0008 (6)	−0.0092 (6)
C19	0.0311 (17)	0.0329 (10)	0.0428 (12)	−0.0115 (9)	−0.0008 (11)	−0.0178 (8)
C20	0.0252 (13)	0.0373 (12)	0.0430 (12)	−0.0120 (10)	0.0024 (9)	−0.0196 (10)
C21	0.044 (2)	0.0553 (15)	0.0303 (14)	−0.0316 (14)	0.0065 (11)	−0.0203 (13)
C19'	0.0311 (17)	0.0329 (10)	0.0428 (12)	−0.0115 (9)	−0.0008 (11)	−0.0178 (8)
C20'	0.0252 (13)	0.0373 (12)	0.0430 (12)	−0.0120 (10)	0.0024 (9)	−0.0196 (10)
C21'	0.044 (2)	0.0553 (15)	0.0303 (14)	−0.0316 (14)	0.0065 (11)	−0.0203 (13)
C22	0.0419 (11)	0.0465 (11)	0.0282 (8)	−0.0277 (9)	0.0029 (7)	−0.0130 (7)
C23	0.0269 (9)	0.0277 (8)	0.0269 (7)	−0.0165 (7)	0.0010 (6)	−0.0066 (6)
C24	0.0295 (10)	0.0215 (8)	0.0295 (8)	−0.0074 (7)	−0.0027 (7)	−0.0032 (6)
C25	0.0276 (9)	0.0224 (8)	0.0238 (7)	−0.0095 (7)	−0.0026 (6)	−0.0016 (6)
C26	0.0238 (9)	0.0211 (7)	0.0253 (7)	−0.0111 (7)	−0.0031 (6)	−0.0019 (6)
C27	0.0237 (9)	0.0210 (8)	0.0279 (7)	−0.0120 (7)	−0.0049 (6)	0.0001 (6)
C28	0.0298 (10)	0.0289 (8)	0.0234 (7)	−0.0156 (8)	−0.0035 (6)	−0.0027 (6)
C29	0.0216 (9)	0.0226 (8)	0.0283 (7)	−0.0115 (7)	−0.0010 (6)	−0.0035 (6)
C30	0.0243 (9)	0.0181 (7)	0.0262 (7)	−0.0052 (7)	0.0015 (6)	−0.0046 (6)
C31	0.0313 (10)	0.0297 (9)	0.0340 (8)	−0.0141 (8)	0.0016 (7)	−0.0048 (7)
C32	0.0302 (10)	0.0326 (9)	0.0367 (9)	−0.0140 (8)	0.0072 (7)	−0.0022 (7)
C33	0.0329 (11)	0.0312 (9)	0.0255 (8)	−0.0073 (8)	0.0050 (7)	0.0007 (6)
C34	0.0279 (10)	0.0282 (8)	0.0265 (7)	−0.0061 (8)	0.0009 (7)	−0.0073 (6)
C35	0.0245 (9)	0.0195 (7)	0.0244 (7)	−0.0053 (7)	0.0033 (6)	−0.0062 (6)
C36	0.0238 (9)	0.0209 (7)	0.0240 (7)	−0.0046 (7)	0.0011 (6)	−0.0078 (6)

### Geometric parameters (Å, °)

**Table:**

O1—C9	1.3506 (18)	C15—H15	0.9500
O1—H1	0.956 (18)	C16—C17	1.388 (2)
O2—C11	1.2531 (18)	C16—H16	0.9500
O3—C18	1.2635 (18)	C17—C18	1.488 (2)
O4—C18	1.2754 (18)	C19—C20	1.528 (3)
O4—H4	0.879 (10)	C19—H19A	0.9900
O5—C27	1.3535 (18)	C19—H19B	0.9900
O5—H5	0.943 (19)	C20—C21	1.529 (3)
O6—C29	1.2419 (18)	C20—H20A	0.9900
O7—C36	1.2675 (18)	C20—H20B	0.9900
O7—H7A	0.86 (2)	C21—C22	1.528 (3)
O8—C36	1.2708 (18)	C21—H21A	0.9900
N1—C5	1.3577 (19)	C21—H21B	0.9900
N1—C4	1.461 (2)	C19'—C20'	1.532 (10)
N1—C1	1.470 (2)	C19'—H19C	0.9900
N2—C23	1.350 (2)	C19'—H19D	0.9900
N2—C22	1.464 (2)	C20'—C21'	1.512 (9)
N2—C19	1.464 (3)	C20'—H20C	0.9900
N2—C19'	1.481 (10)	C20'—H20D	0.9900
C1—C2	1.528 (3)	C21'—C22	1.517 (9)
C1—H1A	0.9900	C21'—H21C	0.9900
C1—H1B	0.9900	C21'—H21D	0.9900
C2—C3	1.514 (3)	C22—H22A	0.9600
C2—H2A	0.9900	C22—H22B	0.9600
C2—H2B	0.9900	C22—H22C	0.9600
C3—C4	1.523 (2)	C22—H22D	0.9600
C3—H3A	0.9900	C23—C28	1.404 (2)
C3—H3B	0.9900	C23—C24	1.427 (2)
C4—H4A	0.9900	C24—C25	1.357 (2)
C4—H4B	0.9900	C24—H24	0.9500
C5—C10	1.410 (2)	C25—C26	1.416 (2)
C5—C6	1.419 (2)	C25—H25	0.9500
C6—C7	1.366 (2)	C26—C27	1.422 (2)
C6—H6	0.9500	C26—C29	1.439 (2)
C7—C8	1.407 (2)	C27—C28	1.374 (2)
C7—H7	0.9500	C28—H28	0.9500
C8—C9	1.419 (2)	C29—C30	1.513 (2)
C8—C11	1.445 (2)	C30—C31	1.393 (2)
C9—C10	1.383 (2)	C30—C35	1.398 (2)
C10—H10	0.9500	C31—C32	1.387 (2)
C11—C12	1.505 (2)	C31—H31	0.9500
C12—C13	1.395 (2)	C32—C33	1.378 (2)
C12—C17	1.404 (2)	C32—H32	0.9500
C13—C14	1.390 (2)	C33—C34	1.391 (2)
C13—H13	0.9500	C33—H33	0.9500
C14—C15	1.379 (3)	C34—C35	1.401 (2)
C14—H14	0.9500	C34—H34	0.9500
C15—C16	1.389 (2)	C35—C36	1.486 (2)
			
C9—O1—H1	105.3 (12)	C19—C20—H20B	111.3
C18—O4—H4	108.2 (14)	C21—C20—H20B	111.3
C27—O5—H5	103.5 (12)	H20A—C20—H20B	109.2
C36—O7—H7A	113.3 (12)	C22—C21—C20	106.09 (19)
C5—N1—C4	123.71 (13)	C22—C21—H21A	110.5
C5—N1—C1	123.89 (13)	C20—C21—H21A	110.5
C4—N1—C1	112.20 (12)	C22—C21—H21B	110.5
C23—N2—C22	123.22 (14)	C20—C21—H21B	110.5
C23—N2—C19	123.37 (16)	H21A—C21—H21B	108.7
C22—N2—C19	113.35 (16)	N2—C19'—C20'	99.6 (9)
C23—N2—C19'	130.8 (7)	N2—C19'—H19C	111.9
C22—N2—C19'	105.2 (8)	C20'—C19'—H19C	111.9
N1—C1—C2	102.82 (14)	N2—C19'—H19D	111.9
N1—C1—H1A	111.2	C20'—C19'—H19D	111.9
C2—C1—H1A	111.2	H19C—C19'—H19D	109.6
N1—C1—H1B	111.2	C21'—C20'—C19'	113.5 (14)
C2—C1—H1B	111.2	C21'—C20'—H20C	108.9
H1A—C1—H1B	109.1	C19'—C20'—H20C	108.9
C3—C2—C1	102.90 (16)	C21'—C20'—H20D	108.9
C3—C2—H2A	111.2	C19'—C20'—H20D	108.9
C1—C2—H2A	111.2	H20C—C20'—H20D	107.7
C3—C2—H2B	111.2	C20'—C21'—C22	92.4 (8)
C1—C2—H2B	111.2	C20'—C21'—H21C	113.2
H2A—C2—H2B	109.1	C22—C21'—H21C	113.2
C2—C3—C4	103.74 (14)	C20'—C21'—H21D	113.2
C2—C3—H3A	111.0	C22—C21'—H21D	113.2
C4—C3—H3A	111.0	H21C—C21'—H21D	110.6
C2—C3—H3B	111.0	N2—C22—C21'	117.1 (6)
C4—C3—H3B	111.0	N2—C22—C21	102.47 (16)
H3A—C3—H3B	109.0	N2—C22—H22A	111.2
N1—C4—C3	103.05 (13)	C21'—C22—H22A	111.1
N1—C4—H4A	111.2	C21—C22—H22A	111.3
C3—C4—H4A	111.2	N2—C22—H22B	111.3
N1—C4—H4B	111.2	C21'—C22—H22B	95.7
C3—C4—H4B	111.2	C21—C22—H22B	111.3
H4A—C4—H4B	109.1	H22A—C22—H22B	109.2
N1—C5—C10	120.69 (14)	N2—C22—H22C	108.0
N1—C5—C6	120.76 (14)	C21'—C22—H22C	108.0
C10—C5—C6	118.54 (14)	C21—C22—H22C	106.1
C7—C6—C5	120.17 (14)	H22B—C22—H22C	116.6
C7—C6—H6	119.9	N2—C22—H22D	108.0
C5—C6—H6	119.9	C21'—C22—H22D	108.0
C6—C7—C8	122.66 (14)	C21—C22—H22D	124.1
C6—C7—H7	118.7	H22A—C22—H22D	99.8
C8—C7—H7	118.7	H22C—C22—H22D	107.3
C7—C8—C9	116.69 (13)	N2—C23—C28	121.15 (14)
C7—C8—C11	122.95 (14)	N2—C23—C24	120.63 (15)
C9—C8—C11	120.32 (13)	C28—C23—C24	118.22 (14)
O1—C9—C10	117.73 (13)	C25—C24—C23	120.37 (15)
O1—C9—C8	120.52 (13)	C25—C24—H24	119.8
C10—C9—C8	121.75 (14)	C23—C24—H24	119.8
C9—C10—C5	120.19 (14)	C24—C25—C26	122.33 (14)
C9—C10—H10	119.9	C24—C25—H25	118.8
C5—C10—H10	119.9	C26—C25—H25	118.8
O2—C11—C8	121.94 (14)	C25—C26—C27	116.66 (14)
O2—C11—C12	116.45 (13)	C25—C26—C29	122.93 (13)
C8—C11—C12	121.58 (13)	C27—C26—C29	120.41 (14)
C13—C12—C17	118.87 (14)	O5—C27—C28	118.40 (13)
C13—C12—C11	119.66 (15)	O5—C27—C26	120.04 (14)
C17—C12—C11	120.91 (13)	C28—C27—C26	121.56 (14)
C14—C13—C12	120.46 (16)	C27—C28—C23	120.77 (13)
C14—C13—H13	119.8	C27—C28—H28	119.6
C12—C13—H13	119.8	C23—C28—H28	119.6
C15—C14—C13	120.30 (16)	O6—C29—C26	123.17 (13)
C15—C14—H14	119.9	O6—C29—C30	118.04 (14)
C13—C14—H14	119.9	C26—C29—C30	118.60 (13)
C14—C15—C16	119.98 (15)	C31—C30—C35	119.40 (14)
C14—C15—H15	120.0	C31—C30—C29	116.62 (14)
C16—C15—H15	120.0	C35—C30—C29	123.87 (13)
C17—C16—C15	120.23 (16)	C32—C31—C30	120.88 (16)
C17—C16—H16	119.9	C32—C31—H31	119.6
C15—C16—H16	119.9	C30—C31—H31	119.6
C16—C17—C12	120.14 (15)	C33—C32—C31	119.93 (16)
C16—C17—C18	117.52 (15)	C33—C32—H32	120.0
C12—C17—C18	122.18 (13)	C31—C32—H32	120.0
O3—C18—O4	123.47 (15)	C32—C33—C34	120.08 (15)
O3—C18—C17	118.89 (13)	C32—C33—H33	120.0
O4—C18—C17	117.57 (13)	C34—C33—H33	120.0
N2—C19—C20	103.8 (2)	C33—C34—C35	120.42 (15)
N2—C19—H19A	111.0	C33—C34—H34	119.8
C20—C19—H19A	111.0	C35—C34—H34	119.8
N2—C19—H19B	111.0	C30—C35—C34	119.28 (14)
C20—C19—H19B	111.0	C30—C35—C36	122.17 (13)
H19A—C19—H19B	109.0	C34—C35—C36	118.49 (14)
C19—C20—C21	102.5 (2)	O7—C36—O8	123.24 (15)
C19—C20—H20A	111.3	O7—C36—C35	118.03 (13)
C21—C20—H20A	111.3	O8—C36—C35	118.71 (13)
			
C5—N1—C1—C2	161.65 (17)	N2—C19'—C20'—C21'	−37 (3)
C4—N1—C1—C2	−13.4 (2)	C19'—C20'—C21'—C22	33 (2)
N1—C1—C2—C3	32.2 (2)	C23—N2—C22—C21'	−172.6 (10)
C1—C2—C3—C4	−39.5 (2)	C19—N2—C22—C21'	4.6 (10)
C5—N1—C4—C3	174.12 (16)	C19'—N2—C22—C21'	−1.7 (18)
C1—N1—C4—C3	−10.8 (2)	C23—N2—C22—C21	177.07 (18)
C2—C3—C4—N1	31.0 (2)	C19—N2—C22—C21	−5.8 (3)
C4—N1—C5—C10	1.2 (2)	C19'—N2—C22—C21	−12.1 (15)
C1—N1—C5—C10	−173.29 (16)	C20'—C21'—C22—N2	−18.3 (17)
C4—N1—C5—C6	−179.87 (16)	C20'—C21'—C22—C21	17 (2)
C1—N1—C5—C6	5.6 (3)	C20—C21—C22—N2	25.2 (2)
N1—C5—C6—C7	−178.58 (15)	C20—C21—C22—C21'	−123 (3)
C10—C5—C6—C7	0.3 (2)	C22—N2—C23—C28	−1.5 (2)
C5—C6—C7—C8	0.1 (2)	C19—N2—C23—C28	−178.4 (3)
C6—C7—C8—C9	−0.9 (2)	C19'—N2—C23—C28	−170 (2)
C6—C7—C8—C11	−178.70 (15)	C22—N2—C23—C24	178.59 (15)
C7—C8—C9—O1	−178.27 (13)	C19—N2—C23—C24	1.7 (3)
C11—C8—C9—O1	−0.4 (2)	C19'—N2—C23—C24	10 (2)
C7—C8—C9—C10	1.2 (2)	N2—C23—C24—C25	−177.27 (15)
C11—C8—C9—C10	179.11 (14)	C28—C23—C24—C25	2.8 (2)
O1—C9—C10—C5	178.70 (14)	C23—C24—C25—C26	−1.1 (2)
C8—C9—C10—C5	−0.8 (2)	C24—C25—C26—C27	−1.5 (2)
N1—C5—C10—C9	178.92 (14)	C24—C25—C26—C29	179.25 (15)
C6—C5—C10—C9	0.0 (2)	C25—C26—C27—O5	−177.00 (14)
C7—C8—C11—O2	177.58 (14)	C29—C26—C27—O5	2.2 (2)
C9—C8—C11—O2	−0.2 (2)	C25—C26—C27—C28	2.5 (2)
C7—C8—C11—C12	−4.7 (2)	C29—C26—C27—C28	−178.22 (14)
C9—C8—C11—C12	177.58 (14)	O5—C27—C28—C23	178.68 (14)
O2—C11—C12—C13	120.26 (16)	C26—C27—C28—C23	−0.9 (2)
C8—C11—C12—C13	−57.6 (2)	N2—C23—C28—C27	178.27 (14)
O2—C11—C12—C17	−51.0 (2)	C24—C23—C28—C27	−1.8 (2)
C8—C11—C12—C17	131.09 (16)	C25—C26—C29—O6	175.12 (15)
C17—C12—C13—C14	0.4 (2)	C27—C26—C29—O6	−4.1 (2)
C11—C12—C13—C14	−171.03 (14)	C25—C26—C29—C30	−10.0 (2)
C12—C13—C14—C15	0.9 (2)	C27—C26—C29—C30	170.85 (14)
C13—C14—C15—C16	−1.4 (3)	O6—C29—C30—C31	110.01 (17)
C14—C15—C16—C17	0.5 (2)	C26—C29—C30—C31	−65.2 (2)
C15—C16—C17—C12	0.8 (2)	O6—C29—C30—C35	−66.1 (2)
C15—C16—C17—C18	−174.64 (15)	C26—C29—C30—C35	118.69 (17)
C13—C12—C17—C16	−1.3 (2)	C35—C30—C31—C32	0.7 (2)
C11—C12—C17—C16	170.06 (14)	C29—C30—C31—C32	−175.58 (15)
C13—C12—C17—C18	173.97 (14)	C30—C31—C32—C33	0.3 (3)
C11—C12—C17—C18	−14.7 (2)	C31—C32—C33—C34	−0.7 (3)
C16—C17—C18—O3	141.35 (16)	C32—C33—C34—C35	0.1 (3)
C12—C17—C18—O3	−34.0 (2)	C31—C30—C35—C34	−1.3 (2)
C16—C17—C18—O4	−35.6 (2)	C29—C30—C35—C34	174.75 (15)
C12—C17—C18—O4	149.04 (16)	C31—C30—C35—C36	175.67 (15)
C23—N2—C19—C20	161.5 (2)	C29—C30—C35—C36	−8.3 (2)
C22—N2—C19—C20	−15.6 (4)	C33—C34—C35—C30	0.9 (2)
C19'—N2—C19—C20	22 (7)	C33—C34—C35—C36	−176.20 (15)
N2—C19—C20—C21	30.0 (4)	C30—C35—C36—O7	−16.5 (2)
C19—C20—C21—C22	−34.7 (3)	C34—C35—C36—O7	160.48 (15)
C23—N2—C19'—C20'	−169.3 (7)	C30—C35—C36—O8	165.09 (15)
C22—N2—C19'—C20'	21 (2)	C34—C35—C36—O8	−17.9 (2)
C19—N2—C19'—C20'	−124 (8)		

### Hydrogen-bond geometry (Å, °)

**Table:**

*D*—H···*A*	*D*—H	H···*A*	*D*···*A*	*D*—H···*A*
O1—H1···O2	0.956 (19)	1.66 (2)	2.547 (2)	151.8 (18)
O5—H5···O6	0.943 (19)	1.68 (2)	2.565 (2)	154.9 (19)
O7—H7A···O3^i^	0.86 (2)	1.78 (1)	2.6387 (17)	169 (2)
O4—H4···O8^ii^	0.88 (1)	1.79 (1)	2.6451 (19)	166 (2)

Symmetry codes: (i) *x*+1, *y*, *z*−1; (ii) *x*−1, *y*, *z*+1.
